# The Relation between the Eye and the Female Genital Organs

**Published:** 1894-02

**Authors:** 


THE RELATION BETWEEN THE EYE AND THE FEMALE GENITAL ORGANS.—Boorne Bettman, M. D. (*Am. Journ. Obs.*; Vol. XXVIII., No. 190.)

Until quite recently this subject has engaged the attention of oculists in a desultory manner only, and but few efforts have been made to collect scientific data, although the importance of gynecological diseases as etiological factors in ocular disturbances has long been recognized.

Although an invasion of disease by continuity of tissue is impossible in these cases, there is a direct connection between the two sets of organs by the nervous, vascular and lymphatic systems. Many of the ocular disturbances are of a reflex character, and many are communicated through the vascular system, but the largest proportion are dependent upon the general constitutional conditions having their origin in diseases of the uterus or its appendages. It is the opinion of Knies, and indorsed by the author, that the importance of diseases of woman as the direct cause of ocular disturbances is universally exaggerated.

Menstruation has been held accountable for a variety of ocular lesions, and these lesions may undergo exacerbations at regular monthly intervals. Hemorrhages into the retina and vitreous occur during menstruation, and blepharitis, hordeolum, keratitis and herpes may develop during the periods. Disorders of menstruation may be the direct cause of hemorrhages into the eye or into the sheath of the optic nerve and brain. Severe post-partum hemorrhage may result in amblyopia and amaurosis. Under the name of kopiopia hysterica are described a complexity of symptoms indicating hyperesthesia of the fifth and optic nerves, and attributed to a chronic inflammation of the nerve-bearing peri-uterine tissue. Other reflex optic phenomena known as muscular and accommodating asthenopia are attributed to the same cause.

Retinitis albuminurica may be attributed indirectly to the pressure of the enlarged uterus during the latter half of pregnancy and occurs in about five per cent. of all pregnancies. Long continued vomiting, as that occurring in pregnancy, may produce hemorrhages into the retina or vitreous, under the conjunctiva, into the brain or optic sheath, resulting in hemianopsia, amblyopia, and even amaurosis. During labor mydriasis is frequently observed, and is due to reflex action along the sympathetic nerves.

Metastatic choroiditis is perhaps the severest optical complication of puerperal sepsis, and may result in atrophy of the eyeball or in complete destruction through panophthalmitis.

The only eye disturbances which are regarded as pathognomonic of uterine disease are those reflex troubles called by Förster kyphopia hysterica, but even these have not been verified satisfactorily by later research.—*American Medico-Surgical Bulletin.*

